# Chlorine Not an Element

**Published:** 1881-11

**Authors:** 


### CHLORINE NOT AN ELEMENT.

Professor Meyer of Berne, claims to have discovered that Chlorine is not an element, as has been supposed, but an oxide of metal, to which he gives the name of Murium. His method of proving this is to subject Chlorine to a temperature of 700° C. This separates the oxygen from the element; the oxygen is collected by passing the mixture through a bath of Mercury, and its nature confirmed by the usual tests. The metal Murium formed an amalgam with the Mercury. Not enough of it has been collected to permit any adequate examination of its properties.

